# A Supramolecular Hydrogel Based on Copolymers of Acrylic Acid and Maleic Anhydride Derivatives with Terpyridine Motifs

**DOI:** 10.3390/polym14142857

**Published:** 2022-07-13

**Authors:** Zheng Chi, Chenchen Ma, Ziyuan He, Zihan Ma, Xuegang Chen, Zhaoge Huang

**Affiliations:** Key Laboratory of Rubber-Plastic of Ministry of Education (QUST), School of Polymer Science and Engineering, Qingdao University of Science and Technology, Qingdao 266042, China; cz17854239116@126.com (Z.C.); 19511618633@163.com (C.M.); hzy15309081778@126.com (Z.H.); mazihan20212021@163.com (Z.M.)

**Keywords:** supramolecular hydrogel, acrylic acid, maleic anhydride, terpyridine, coordination interaction

## Abstract

A kind of terpyridine derivative (**NH_2_-Tpy**) in which the amino was incorporated by a short alkyl chain was synthesized. Through grafting of terpyridine units into the hydrophilic copolymers of maleic anhydride and acrylic acid **PAAMa** via the reaction of the amino groups in **NH_2_-Tpy** and the maleic anhydride units, a series of gelator polymers—**P1**, **P2**, and **P3**—containing different contents of terpyridine units was synthesized. Under coordination of Ni^2+^ and terpyridine ligands in linear polymers, the supramolecular hydrogels **H1**, **H2**, and **H3** with different cross-linking degrees were prepared. The linear polymers **P1**–**P3** had a strong absorption peak at about 290 nm in the UV-vis spectra which was attributed to π–π* transition, and there was a new peak at about 335 nm led by the metal-to-ligands charge transfer (MLCT) when coordinated with Ni^2+^ ions. According to the rheological behaviors, the storage modulus (G′) was larger than the loss modulus (G′′). These hydrogels showed typical gel-like characteristics when the terpyridine content of the hydrogels exceeded 10%, and the hydrogels showed liquid-like characteristics when the terpyridine content of the hydrogels was less than 7%. The results of the micromorphological investigation of the xerogels from SEM illustrated the metal–terpyridine coordination cross-linking could have an important influence on the microstructures of the resulting hydrogels. Furthermore, these hydrogels based on supramolecular cross-links exhibited reversible solution–gel transition at different environmental temperatures. At the same time, the equilibrium swelling of the supramolecular hydrogels was 8.0–12.3 g/g, which increased with the decrease in the content of the terpyridine units in the resulting hydrogels.

## 1. Introduction

Hydrogels are polymeric materials with 3D networks of cross-linked structures that can retain a large amount of water in their swollen state through capillary, osmotic, and hydration forces [1,2]. Great advances have been made in supramolecular polymers over the past several decades [3,4]. As one of the most important subfields, supramolecular hydrogels refer to the soft materials that are constructed via non-covalent interactions such as hydrogen bonds, π–π interactions, van der Waals interactions, hydrophobic interactions, ionic bonds, host–guest interactions, metal–ligands coordination interactions, etc., and have attracted great enthusiasm for these fields [5,6,7,8,9]. Supramolecular assembly systems based on multiple, orthogonal non-covalent motifs allow for the preparation of controlled, reversible, selective, multi-stimuli-responsive hydrogel materials, and supramolecular hydrogels play a vital role in biological science, drug transport, tissue engineering, wound dressing, and so on [10,11,12].

Generally, most covalent chemistry is irreversible, whereas most supramolecular non-covalent chemistry is reversible, and thus some thermodynamic behaviors could occur in these systems [13]. Many supramolecular hydrogels have stimulus–response characteristics, especially thermal responsiveness, and metal–ligand-coordination-based supramolecular hydrogels with thermo-responsive properties are promising [9,14,15]. The gel–sol transition temperature of supramolecular hydrogels could be adjusted by controlling the amount and strength of the non-covalent coordination bonds, which allows hydrogels to be more accurately applied in various fields [14]. Furthermore, since the metal–ligands coordination bond can usually be rebuilt after being destroyed in the appropriate environment, a supramolecular hydrogel based on metal–ligands has sometimes been used as a tunable thermosensitive material with reversibility [16].

It is known that 2,2′:6′,2′′-terpyridine (tpy)-based ligands show versatile binding with different divalent and trivalent metal ions to form bis-terpyridine complexes, whereas linear coordination polymers or network polymers are obtained from the coordination of ditopic or polytopic ligands with appropriate metals [17,18,19]. Therefore, the tpy-based building blocks applied in supramolecular chemistry and materials have attracted considerable interest, and the ease of modification in the ligands or polymer chains and the introduction of different metals into molecular structures have endowed these building blocks and resulting supramolecular materials with many interesting functional properties [20,21].

There have been some novel supramolecular hydrogels based on terpyridine-metal coordination in recent years [22,23,24,25]. Yu et al. reported a ditopic ligand comprising terpyridine and acetylene segments, and the fluorescence enhancement in the blue emission in solution or gel could occur when coordinated with zinc ions [22]. Yang et al. designed interpenetrating polymer network hydrogels based on metal–terpyridine coordination and dynamic covalent bonds, and the properties of these hydrogels can be adjusted via tunable metal–ligand interaction [23]. The modification of building blocks and the introduction of different metals supply more ways to optimize the properties of these novel supramolecular hydrogels.

Hydrogels originating from the hydrophilic group have good hydrophilicity. Obviously, terpyridine and some functional segments generally show hydrophobic properties, and it is difficult to form hydrogel systems for terpyridine alone. Linking with hydrophilic structures is a possible way to obtain hydrogels for terpyridine–metal complexes. Due to large steric hindrance, it is difficult to form the homo-polymer of maleic anhydride; however, copolymerization based on maleic anhydride is very common, and many copolymers containing maleic anhydride have been prepared and applied in scale inhibition and dispersing agents. As one of the hydrophilic monomers, maleic anhydride (MA) is often used in the preparation of hydrogels [26,27,28]. Furthermore, MA is easy to modify and functionalize for the copolymers containing maleic anhydride through the reaction of acid anhydride groups and other functional groups [29,30,31].

**Scheme 1:**
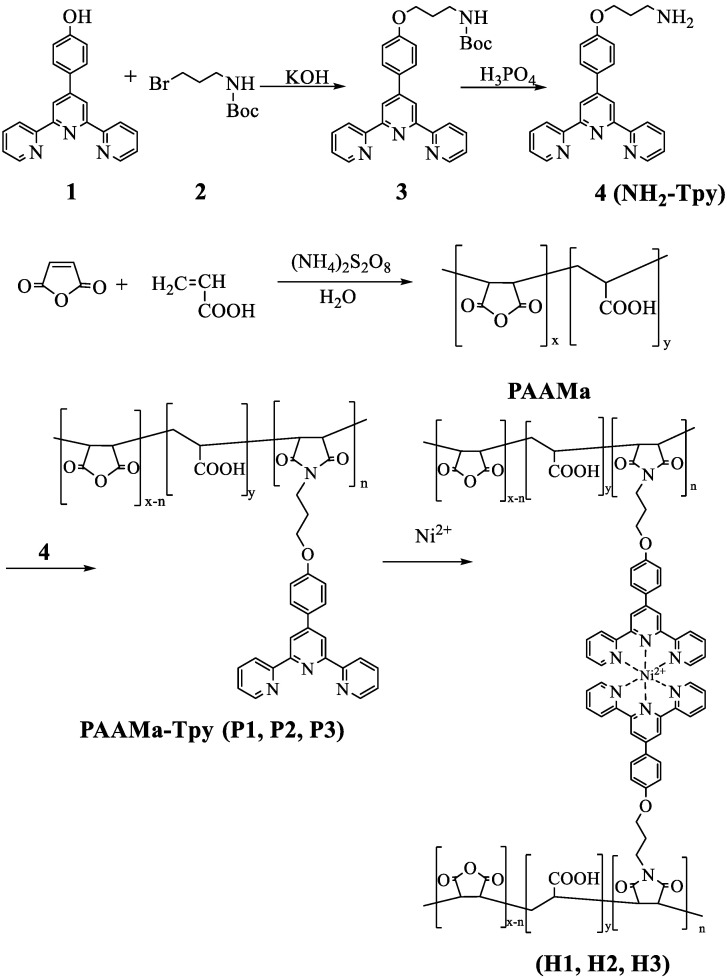
Synthetic routes of the copolymer **PAAMa** and the terpyridine-containing polymers **P1**, **P2**, and **P3**, and the preparation of metallo-supramolecular hydrogels **H1**, **H2,** and **H3**.

Herein, a terpyridine derivative with an amino group (NH_2_-Tpy) was designed and synthesized (Scheme 1). Through the reaction of the amino in NH_2_-Tpy and the anhydride group in a hydrophilic copolymer (PAAMa) of acrylic acid and maleic anhydride prepared from free radical copolymerization, the terpyridine segments were introduced in the copolymers and a series of hydrophilic target gelator polymers (PAAMa-Tpy) were obtained (Scheme 1). The supramolecular hydrogels with different terpyridine contents were prepared via the Ni^2+^–terpyridine coordination bonds formed in aqueous solution. The photophysical properties, morphological characteristics, rheological properties, thermal responsiveness, and the equilibrium swelling ratio of the linear polymers and resulting metallo-supramolecular hydrogels based on non-covalent coordination were studied.

## 2. Materials and Methods

All the chemicals were purchased from commercial sources and used without further purification unless otherwise specified. The 4′-(p-hydrophenyl)-2,2′:6′,2″-terpyridine (**1**) was synthesized via the Kröhnke method according to instructions in [32], 4′-(p-(3-(boc-amine) propoxy)-phenyl)- 2,2′:6′,2″-terpyridine (**3**) was synthesized according to the previous method in [33], and 4′-(p-(3-amine propoxy)-phenyl)- 2,2′:6′,2″-terpyridine (**NH_2_-Tpy**) (4) was synthesized according to the reported method in [34]. The copolymer **PAAMa** was synthesized as indicated in the literature [35]. The gelator polymer (**PAAMa-Tpy**) was synthesized according to the instructions in [36]. The ^1^H NMR spectra and ^13^C NMR spectra were obtained with a Bruker Advance 500 spectrometer. IR spectra were recorded using a Bruker vertex 70 TGA-IR instrument. The UV spectra were recorded with a Hitachi U-4100 instrument. The morphological analysis of all hydrogels was carried out using a JSM-7500F instrument. The viscosity average molecular weight (M_η_) was studied using an Ubbelohde viscometer. The swelling ratio of the resulting supramolecular hydrogel was measured at room temperature. The swelling ratio (g/g) of the sample was calculated: equilibrium swelling ratio (ESR) = (m_t_ − m_0_)/m_0_, where m_0_ and m_t_ are the weights of the xerogel and hydrogel samples at time t, respectively. When a hydrogel reaches its swelling equilibrium state, its swelling ratio is called the equilibrium swelling ratio (ESR). In measurement, the free water should be removed carefully so that precise data can be obtained.

### 2.1. Synthesis of 4′-(p-(3-(boc-amine) propoxy)-phenyl)-2,2′:6′,2″-terpyridine (**3**)

Compound **1** (3.5 g, 10.7 mmol) and (3-(boc-amino)-propyl bromide) (**2**) (3.2 g, 13.4 mmol) were added to the solution of potassium hydroxide (0.7 g, 12.5 mmol) in ethyl alcohol (60 mL) under the protection of nitrogen and stirred for 48 h at 90 °C. After the reaction was completed, the solvent was evaporated, and the residue was separated using column chromatography with PE as eluent. Following this, 4′-(p-(3-(boc-amine) propoxy)-phenyl)- 2,2′:6′,2″-terpyridine (**3**) (4.5 g, 9.3 mmol) was obtained as a white solid with a yield of 87%. ^1^H NMR (CDCl_3_): δ 8.74–8.66 (m, 6H), 7.90–7.88 (m, 4H), 7.37–7.34 (m, 2H), 7.03–7.01 (d, 2H, J = 8.8 Hz), 4.11–4.09 (t, 2H, J = 6.0 Hz), 3.38–3.36 (m, 2H), 2.04–2.01 (m, 2H), 1.46 (s, 9H), 0.98–0.94 (t, 1H, J = 7.4 Hz).

### 2.2. Synthesis of 4′-(p-(3-amine propoxy)-phenyl)-2,2′:6′,2″-terpyridine (NH_2_-Tpy) (**4**)

To the solution of compound **3** (2.0 g, 4.1 mmol) in dichloromethane (15 mL) was added phosphoric acid aqueous solution (85 wt%) (1.5 mL, 7.0 mmol) dropwise; then, the mixture was vigorously stirred at room temperature for 2 h. Sodium hydroxide (0.9 g, 21.0 mmol) aqueous solution (10 mL) was added dropwise at 0 °C until the solvent became weakly alkaline (PH ≈ 8). The solvent was extracted with dichloromethane, and the organic phase was dried with magnesium sulfate and evaporated to obtain **NH_2_-Tpy** as a white solid with a yield of 88%. FT-IR (KBr): 3960.26, 3734.43, 2928.74, 1696.56, 1659.53, 1600.96, 1516.05, 1438.43, 1389.36, 1247.81, 1178.37, 1093.31, 833.34, 788.26, 660.52 cm^−1^. ^1^H NMR (CDCl_3_): δ 8.74–8.66 (m, 6H), 7.90–7.88 (m, 4H), 7.37–7.34 (m, 2H), 7.03–7.01 (d, 2H, J = 8.8 Hz), 4.11–4.08 (t, 2H, J = 6.0 Hz), 3.38–3.36 (m, 2H), 2.96–2.93 (t, 2H, J = 6.6 Hz), 2.04–2.01 (m, 2H). ^13^C NMR (CDCl_3_): 156.4, 155.8, 149.092, 136.8, 128.5, 123.7, 121.4, 118.2, 114.8, 77.3, 77.0, 76.8, 66.0, 39.2, 33.0. 

### 2.3. Synthesis of Copolymer of Acrylic Acid and Maleic Anhydride PAAMa

To the solution of maleic anhydride (10.0 g, 102.0 mmol) in deionized water (30 mL) was added acrylic acid (18.2 g, 252.7 mmol), and the ammonium persulfate water solution (10 mL, 10 wt%). The solvent was stirred for 5 h at 85 °C, and was then cooled to room temperature. The solution was evaporated in a vacuum environment, and the copolymer **PAAMa** was obtained as a white solid with a yield of 96%. M_η_ = 21,000. ^1^H NMR (D_2_O): δ 4.40 (br, 2H), 3.92 (br, 2H), 1.79–1.96 (br, 5H), 1.49–1.50 (br, 10H).

### 2.4. General Synthetic and Purification Procedure of Gelator Polymer

To the three-neck flask was added **NH_2_-Tpy** (2.4 g,6.3 mmol), **PAAMa** (4.5 g), and N,N-dimethylformamide (100 mL) under a nitrogen atmosphere. The mixture was stirred for 48 h at 130 °C, and then the solution was cooled to room temperature. The target gelator polymer **P2** (6.0 g) was obtained as a yellowish solid via precipitation with diethyl ether twice. The other polymers, **P1** and **P3**, with different contents of the terpyridine unit, were prepared with a similar method. 

### 2.5. Preparation of Supramolecular Hydrogels

To the 0.14 mL aqueous solution of NaOH (0.1 g/mL) was added 0.1 g gelator polymer (**P1** or **P2** or **P3**) and 0.86 mL H_2_O. After the polymer was dissolved completely, the 0.5 mL aqueous solution of NiCl_2_·6H_2_O with a concentration of 0.024 g/mL (for **P1**), 0.034 g/mL (for **P2**), and 0.042 g/mL (for **P3**) was added into the polymer solution to obtain supramolecular hydrogels **H1**, **H2**, and **H3**, respectively.

## 3. Results and Discussion

### 3.1. Synthesis of the Hydrophilic Polymers Containing Terpyridine Segments

**Figure 1:**
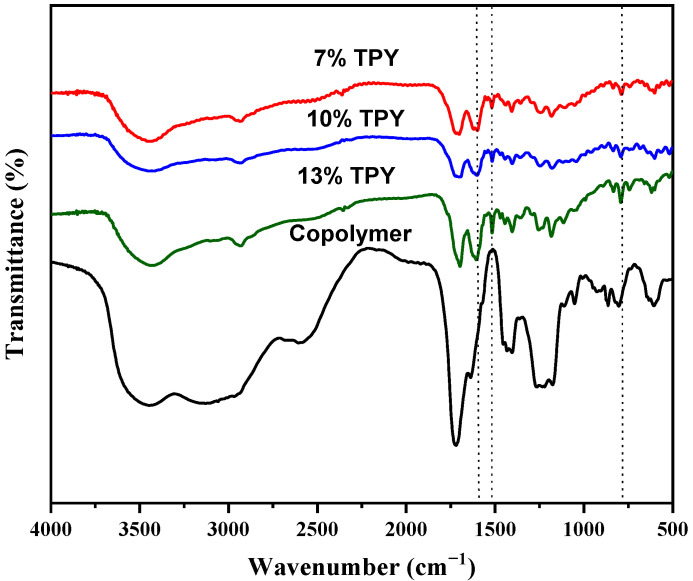
FTIR spectra of **PAAMa**, **P1**, **P2**, and **P3**.

**Figure 2:**
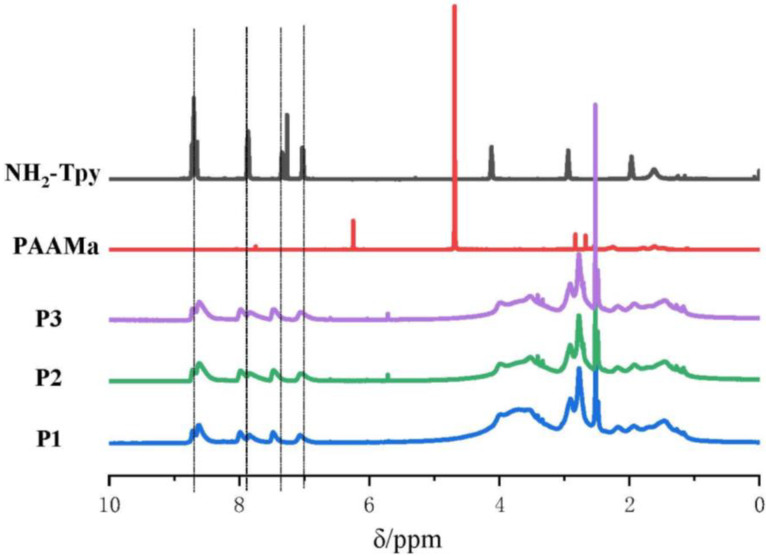
The NMR spectra of **NH_2_-Tpy**, **PAAMa**, and the polymers **P1**, **P2**, and **P3**.

Compound **1** was synthesized via the classic Kröhnke method according to the literature [32]. In an alkaline condition, amino-substituted bromide **2** reacted with the hydroxyl group in compound **1**, and the protected amino functional group could be incorporated on it with a high yield (Scheme 1). Through a deprotection process under an acid condition, the important intermediate **NH_2_-Tpy** can be obtained. The copolymer of acrylic acid and maleic anhydride, **PAAMa**, was simply synthesized via free radical copolymerization of acrylic acid and maleic anhydride initiated by ammonium persulfate. The gelator polymers (**PAAMa-Tpy**) were synthesized via a reaction of different molar ratios of **NH_2_-Tpy** with the polymer **PAAMa** in DMF. Through an amidation process of the terminal amino group in **NH_2_-Tpy** with the anhydride unit in **PAAMa**, the terpyridine segments can be introduced onto **PAAMa** successfully [36]. The resulting linear polymers exhibited good solubility in water due to the large numbers of hydrophilic carboxyl groups from acrylic acid and maleic anhydride. The FTIR spectra of **PAAMa**, **P1**, **P2**, and **P3** are given in Figure 1. It can be seen that the strong stretching vibration absorption peaks of C=O at 1717 cm^−1^ and C-O at 1176 cm^−1^ for **PAAMa** appeared and there were not any aromatic structure signals. The characteristic bands of aromatic skeleton were detected at 1518 cm^−1^, 1595 cm^−1^, and 785 cm^−1^, which were assigned to the aromatic structures in phenyl-substituted 2,2′:6′,2′′-terpyridine in polymers **P1**, **P2**, and **P3**. As seen in Figure 1, the absorption intensity of the aromatic skeleton structures became more and more strong from **P1** to **P3**, which is in agreement with the result of an increase in the terpyridine segments grafted onto the polymer for **P1**-**P3**. The NMR spectroscopy of **NH_2_-Tpy**, **PAAMa**, and three gelator polymers with different terpyridine contents (**P1**, **P2**, and **P3**) are shown in Figure 2. The characteristic peaks of the aromatic part in **NH_2_-Tpy** appeared between 7 and 9 ppm, whereas the **PAAMa** had no signal in this range, and the three polymers had the characteristic peaks of the terpyridine units at the same position as **NH_2_-Tpy**, which indicated that the terpyridine moieties were incorporated onto **PAAMa** successfully.

According to the integrating area of the NMR spectra, the content of the terpyridine segments in the resulting polymers (**PAAMa**) can be calculated, and the results obtained from NMR peaks and the feed percentage of the terpyridine-containing monomer **NH_2_-Tpy** are summarized in Table 1. As shown in Table 1, the feed percentage of **NH_2_-Tpy** was close to the content of terpyridine units calculated from NMR.

### 3.2. Photophysical Properties of the Linear Polymers Containing Terpyridine Segments

**Figure 3:**
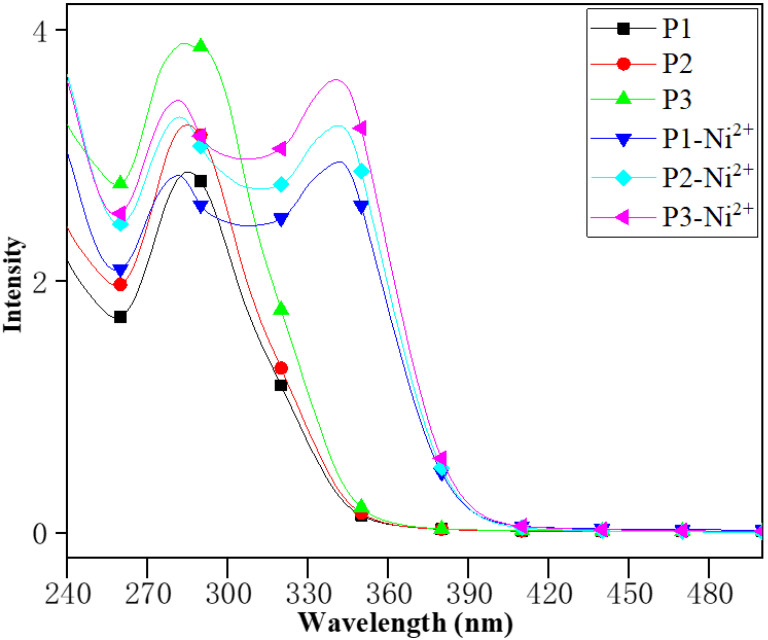
UV spectra of 33 ppm **P1** (7% terpyridine content), **P2** (10% terpyridine content), and **P3** (13% terpyridine content); and 33 ppm **P1**, **P2**, and **P3** with 5 ppm, 7 ppm, and 9 ppm Ni^2+^ in aqueous solution, respectively.

Ultraviolet absorption spectroscopy was used to study the photophysical properties of **PAAMa-Tpy** and the interaction with Ni^2+^ in water solution. As shown in Figure 3, the UV spectra clearly showed that there was an absorption peak at about 290 nm caused by the π–π* transition of the terpyridine-based conjugated structures, and the absorption intensity increased with the increase in the terpyridine content incorporated in the main chains of the resulting polymers [37,38]. An aqueous solution of NiCl_2_·6H_2_O (0.5 mL) with a concentration of 0.0024 g/mL, 0.0034 g/mL, and 0.0042 g/mL was added to an aqueous solution of **P1**, **P2**, and **P3** (9.5 mL) with a concentration of 0.0010 g/mL, respectively. A total of 0.1 mL of the above solution was diluted to 3 mL and tested. In addition to the shorter wavelength peak at about 290 nm similar to that of the uncoordinated polymers, a new absorption peak at about 335 nm appeared, which was attributed to the metal-to-ligand charge transfer (MLCT) after coordination with Ni^2+^, which supports the formation of metal–ligand coordination between terpyridine and Ni^2+^ [24,37,39]. 

### 3.3. Preparation of the Metallo-Supramolecular Hydrogels

**Figure 4:**
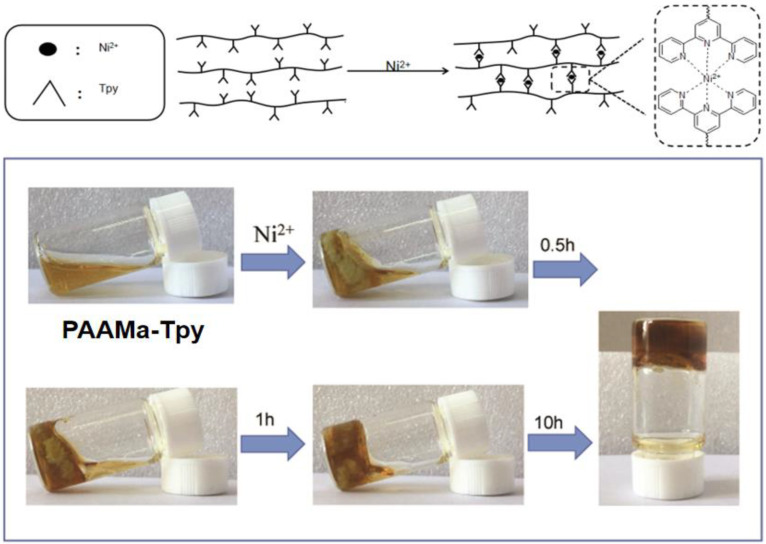
The formation process of supramolecular hydrogel based on the coordination of tpy and Ni^2+^.

As shown in Figure 4, the Ni^2+^ was added to the aqueous solution of **PAAMa-Tpy**. The white complex was first produced as the complex of terpyridine-Ni^2+^ spread in the water; the transparency of the hydrogel then became higher, and finally, a uniform hydrogel was formed. After Ni^2+^ was added, the Ni^2+^ complexed with two terpyridines successively to generate mono-complex Ni(terpyridine)^2+^ and bis-complex Ni(terpyridine)_2_^2+^ to obtain a twisted octahedral structure in which Ni^2+^ was coordinated with six nitrogen atoms [40,41]. The stability constant of bis(terpyridine)-complex Ni(terpyridine)_2_^2+^ can be estimated using the formation rate constant and dissociative rate constant between terpyridine and Ni(terpyridine)^2+^. At 25 °C, the formation rate constant (k_f_) of terpyridine and Ni(terpyridine)^2+^ in water is 10^5.3^ M^−1^·s^−1^, and the dissociative rate constant (k_d_) of Ni(terpyridine)_2_^2+^ is 10^−5.8^ s^−1^. Since the stability constant of Ni(terpyridine)_2_^2+^ K_2_ = k_f_/k_d_ = 10^11.1^ M^−1^ and the stability constant of Ni(terpyridine)^2+^ K_1_ = 10^10.7^ M^−1^, the Ni(terpyridine)_2_^2+^ can exist stably at room temperature [42].

### 3.4. Micromorphological Study

**Figure 5:**
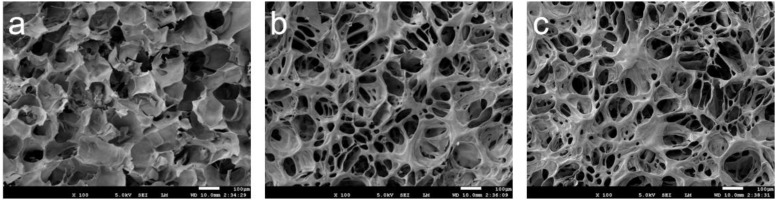
SEM images of (**a**) **H1** (7% terpyridine content), (**b**) **H2** (10% terpyridine content), (**c**) **H3** (13% terpyridine content).

To study the three-dimensional cross-linked networks inside the hydrogels, the microstructures of the xerogels prepared from the corresponding hydrogels via freeze drying were investigated through SEM. As shown in Figure 5a, the pores of the xerogel with 7% terpyridine content were irregular, and there was no molded cross-linked structure inside the hydrogel for **H1**. The partial collapse of the pores probably resulted from the fact that there was no effective or strong cross-linking structure for the low content of the coordination bonds. Figure 5b shows that the xerogel with 10% terpyridine content (**H2**) had uniform pore distribution and a regular structure, and the average diameter of the pores inside the xerogel was about 50–60 μm. Compared with **H1**, the increase in the content of terpyridine-unit-coordinated structures consequently led to a higher cross-linking degree and strength. The results indicate that there was a critical value of about 7–10% for the content of terpyridine units in forming effective cross-linking structures for these coordination-leading supramolecular hydrogels. As Figure 5c shows, the average diameter of the pores in the xerogel with 13% terpyridine content (**H3**) was slightly smaller than that of **H2** (about 50 μm). There were many nanofibers in xerogels **H2** and **H3**, whereas there were almost no nanofiber structures in **H1**. With increasing terpyridine content, the number of Ni^2+^–terpyridine coordination bonds increased; these play a vital role in the formation of 3D network structures and their strength [43,44].

### 3.5. Rheological Properties

**Figure 6:**
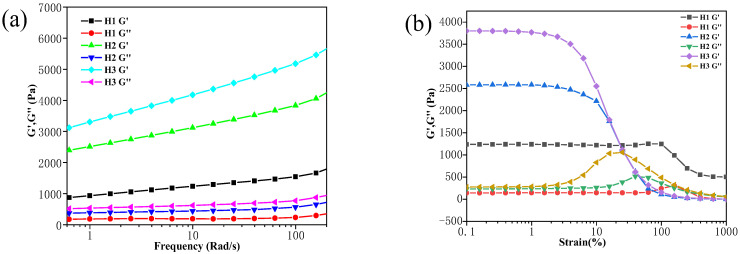
(**a**) Frequency sweep of three hydrogels with **H1** (7% terpyridine content), **H2** (10% terpyridine content), and **H3** (13% terpyridine content); (**b**) strain sweep of **H1**, **H2**, and **H3**.

The rheological properties of the three resulting metallo-supramolecular hydrogels with different terpyridine contents—**H1**, **H2**, and **H3**—with a water content of 90% at 25 °C were studied (Figure 6). As shown in Figure 6a, the changes in G′ (storage moduli) and G″ (loss moduli) of the hydrogels **H1**, **H2**, and **H3** as a function of applied frequency (0–100 Hz) were monitored. Both G′ and G″ increased obviously with increasing terpyridine content in hydrogels, from **H1**, **H2**, to **H3**, which was due to the greater number of metallo-supramolecular cross-link structures that could be formed with the higher content of terpyridine units in hydrogels [45]. At the same time, from 0 to 100 rad/s, both G′ and G″ increased with the increase in frequency and exhibited shear-thinning behaviors, which was attributed to the deformation of the material under shearing forces and the reorganization of the internal structures of the supramolecular hydrogels that appeared [46]. For hydrogel **H1**, with a lower terpyridine content, however, the G′ increased slightly with the increase in frequency and the differentials of G′ and G″ were rather lower at different frequency ranges, most likely because the supramolecular cross-link degree was very low and the networks had not been formed effectively. For all hydrogels, the G′ values were much larger than the corresponding G″, and the gaps between G′ and G″ of hydrogels with 7% (**H1**), 10% (**H2**), and 13% (**H3**) terpyridine content were about 700, 2000, and 2600 Pa, respectively, which were characteristic of an effective cross-linked network. Obviously, the coordination interaction of terpyridine ligands incorporated into the hydrophilic polymer chains with Ni^2+^ drove the formation of a viscoelastic 3D network, and consequently as the terpyridine content in hydrogels increased and then the supramolecular cross-link degree increased, the hydrogels became more solid-like. 

Strain sweep measurements for **H1**, **H2**, and **H3** at a frequency of 6.28 Hz were carried out and the results were as shown in Figure 6b. As the strain increased, the G′ of **H3** decreased sharply, which was caused by the destruction of the Ni^2+^–terpyridine coordination interactions in the hydrogel, and when the strain reached 20%, the curve of G″ appeared at a peak and was crossed over with G′ at this point, which showed that the internal cross-linked networks of the hydrogel were completely destroyed here [47,48]. The shapes of the G′ and G″ curves of the hydrogel **H2** were similar to those of the hydrogel **H3**, but the intersection point of the G′ and G″ curves for **H3** was at 1000 Pa, whereas the intersection point of the hydrogel **H2** was at 500 Pa, which was due to **H3** possessing more terpyridine and more coordination bonds [49]. The G″ peak of **H1** appeared at 250 Pa, which was lower than that of **H2**. By contrast, there was no intersection point between the G′ and G″ of **H1**, mostly due to the low content of terpyridine in the hydrogel **H1**, and there was not enough effective coordination for interaction-based cross-linked structures to be formed in this hydrogel.

### 3.6. Thermo-Responsive Properties

**Figure 7:**
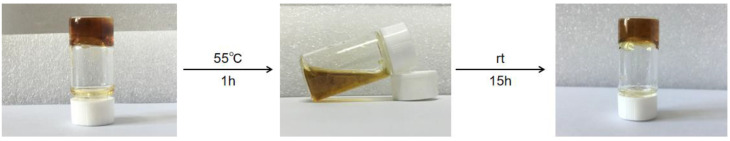
The gel–sol–gel transition recycle of **H1**.

These polymer hydrogels based on metallo-supramolecular cross-linking exhibited interesting reversible sol–gel transition properties at different temperatures. To elucidate the relationship of the sol–gel transition temperature T_gel–sol_ and the internal structure of the hydrogels, the T_gel–sol_ of the hydrogels with different terpyridine contents and different water contents was investigated. As shown in Figure 7, the hydrogel **H1** gradually entered into a homogeneous solution state at about 55 °C for 1 h, and when the environmental temperature decreased to room temperature, the solution turned back to a gel state again slowly. This was probably caused by the disintegration of the Ni^2+^–terpyridine coordination bonds in polymer hydrogels at the temperature above T_gel–sol_, which were rebuilt at room temperature [50]. As shown in Table 2, the T_gel–sol_ of the hydrogels **H1**, **H2**, and **H3** were 55 °C, 60 °C, and 75 °C, respectively. Although there existed equilibrium of free Tpy and Ni(II)(Tpy)_2_ complexes, the equilibrium constant is related to temperature. When the temperature increased, the content of Ni(II)(Tpy)_2_ was reduced to the critical value, and the number of cross-linking bonds was not high enough to keep hydrogels, the gel-to-sol transition occurred. Therefore, the hydrogel with a higher content of terpyridine-Ni(II) coordination bonds (H3) reduced to the critical content at higher temperature. Obviously, the increased cross-linking degree of the polymer hydrogels resulting from the greater number of Ni^2+^–terpyridine coordination bonds could have increased their thermal stability and exhibited a higher T_gel–sol_ [9]. Furthermore, the T_gel–sol_ values of the hydrogel **H3** with water contents of 91%, 90%, and 85% were 70 °C, 75 °C, and 93 °C, respectively. The thermal stability of the hydrogels increased with the increase in the gelator content because of the increased intermolecular interactions and network strength [51,52]. After heating and cooling the same sample three times, the hydrogel still showed good phase transition characteristics, which indicates that the supramolecular hydrogels have an excellent dynamic reversible character.

### 3.7. Swelling Behavior

**Figure 8:**
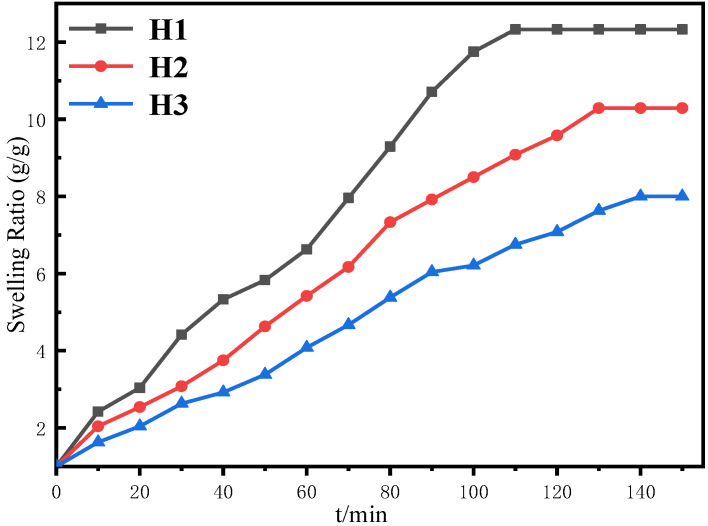
The equilibrium swelling ratio of the supramolecular polymer hydrogels **H1**, **H2**, and **H3**.

These hydrogels contain a large number of hydrophilic groups in the main chains and exhibited water absorption properties. The swelling ratios of the metallo-supramolecular hydrogels **H1**, **H2**, and **H3** were measured at room temperature when a hydrogel reached its swelling equilibrium state. The results are shown in Figure 8. Generally, the water absorption ratio of the cross-linking polymer hydrogel decreased with increasing cross-linking degree of the hydrogel. As shown in Figure 8, the equilibrium swelling ratio of the resulting supramolecular hydrogels **H1**, **H2**, and **H3** were 12.3 g/g, 10.3 g/g, and 8.0 g/g, respectively. These results were caused by the different cross-linking degrees for different hydrogels. The cross-linking of these metallo-supramolecular hydrogels was obtained via coordination interactions between metal ions and terpyridine units incorporated in the linear polymer backbones. The content of terpyridine units in **P1**, **P2**, and **P3** were 7.0%, 10.0%, and 13.0%, respectively, and consequently the cross-linking degrees for the resulting hydrogels are in the following order: **H1** < **H2** < **H3**. Fewer terpyridine units in hydrophilic polymers led to fewer coordination bonds and a lower cross-linking degree in the hydrogels. The cross-linking network can swell more sufficiently, and more water can enter into the network and bind with the hydrophilic polymer structures. Consequently, the swelling ratio order for these supramolecular hydrogels was: **H1** > **H2** > **H3**. These supramolecular hydrogels showed high water-absorption speed, and the times to reach the swelling equilibrium state were 110 min, 130 min, and 140 min for **H1**, **H2**, and **H3**, respectively. Obviously, the low cross-linking degree was beneficial for the diffusion into the 3D hydrophilic network.

## 4. Conclusions

A novel metallo-supramolecular hydrogel was developed. The hydrophilic polymers were based on acrylic acid and maleic anhydride, and used radical polymerization. The gelator polymers containing different terpyridine units were obtained through grafting reactions and the NMR results indicate that the synthesis was successful. The metallo-supramolecular hydrogels were prepared via a coordination interaction of the terpyridine units and metal ions, and the photophysical properties, rheological properties, micromorphology, thermo-responsive properties, and equilibrium swelling behaviors were fully studied. The graft ratio of the terpyridine unit onto the main chains of the hydrophilic copolymers of acrylic acid and maleic anhydride can be tuned easily. The content of the ligand unit terpyridine played a vital role in the microstructures, mechanical performances, and equilibrium swelling ratio due to the fact that the cross-linking degree can be controlled conveniently via changing the content of terpyridine units in raw linear hydrophilic polymers. The interesting thermal-responsive characteristics originated from the non-covalent cross-linking bonds, and these can be further used as intelligent polymer hydrogels. In general, these hydrogels based on coordination show good promise for environmental responses, biosensors, and similar. In the future, adhesives, biocompatibility, photo-function, and so on based on different back-bone systems should be investigated and their application widened.

## Figures and Tables

**Table 1:** The feed content of monomer **NH_2_-Tpy**, and the content of terpyridine segments calculated from the NMR integrating area and the conversion rate of the radical polymerization for **P1**, **P2**, and **P3**.

Polymer	Feed Percentage of NH_2_-Tpy (%)	Terpyridine Content from NMR (%)	Conversion Rate (%)
**P1**	7.2	6.9	95.8
**P2**	10.4	10.1	97.1
**P3**	13.5	12.7	94.1

**Table 2:** The transition temperature of **H1**, **H2**, and **H3** with 90% water content.

Hydrogel	Transition Temperature
**H1**	55 °C
**H2**	60 °C
**H3**	75 °C

## Data Availability

No statement.

## References

[1] Ahmed E.M. (2015). Hydrogel: Preparation, Characterization, and Applications: A review. J. Adv. Res..

[2] Hu W.K., Wang Z.J., Xiao Y., Zhang S.M., Wang J.L. (2019). Advances in Crosslinking Strategies of Biomedical Hydrogels. Biomater. Sci..

[3] Brunsveld L., Folmer B.J.B.E., Meijer W., Sijbesma R.P. (2001). Supramolecular Polymers. Chem. Rev..

[4] Li S.L., Xiao T.X., Lin C., Wang L.Y. (2012). Advanced Supramolecular Polymers Constructed by Orthogonal Self-Assembly. Chem. Soc. Rev..

[5] Hapiot F., Menuel S., Monflier E. (2013). Thermoresponsive Hydrogels in Catalysis. ACS Catal..

[6] Ma X., Tian H. (2014). Stimuli-Responsive Supramolecular Polymers in Aqueous Solution. Acc. Chem. Res..

[7] Shigemitsu H., Hamachi I. (2017). Design Strategies of Stimuli-Responsive Supramolecular Hydrogels Relying on Structural Analyses and Cell-Mimicking Approaches. Acc. Chem. Res..

[8] Shao T., Falcone N., Kraatz H.B. (2020). Supramolecular Peptide Gels: Influencing Properties by Metal Ion Coordination and Their Wide-Ranging Applications. ACS Omega.

[9] Nandi N., Gayen K., Ghosh S., Bhunia D., Kirkham S., Sen S.K., Ghosh S., Hamley I.W., Banerjee A. (2017). Amphiphilic Peptide-based Supramolecular, Non-Cytotoxic Stimuli-Responsive Hydrogels with Antibacterial Activity. Biomacromolecules.

[10] Hymes J., Wolf B. (1996). Biotinidase and Its Role in Biotin Metabolism. Clin. Chim. Acta.

[11] Daigo M., Fumito A., Hideo T., Jungeun K., Katomasaki T., Takuzo A. (2012). Ferroelectric Columnar Liquid Crystal Featuring Confined Polar Groups Within Core-Shell Architecture. Science.

[12] Santanu B., Yamuna K.G. (2001). First Report of Phase Selective Gelation of Oil from Oil/Water Mixtures. Possible Implications Toward Containing Oil Spills. Chem. Commun..

[13] Rowan S.J., Cantril S.J., Cousins G.R.L., Jeremy K.M., Sanders J.K.M., Stoddart J.F. (2010). Dynamic Covalent Chemistry. Angew. Chem. Int. Ed. Engl..

[14] Sun Z.F., Li Z.Y., He Y.H., Shen R.J., Deng L., Yang M.H., Liang Y.Z., Zhang Y. (2013). Ferrocenoyl Phenylalanine: A New Strategy Toward Supramolecular Hydrogels with Multistimuli Responsive Properties. J. Am. Chem. Soc..

[15] Zhao X.J., Wang Q., Du M. (2007). Metal-Directed Assembly of 1-D and 2-D Coordination Polymers with Fluconazole and Dicyanamide Co-Ligand. Inorg. Chim. Acta.

[16] Fullenkamp D.E., He L.H., Barrett D.G., Burghardt W.R., Messersmith P.B. (2013). Mussel-Inspired Histidine-Based Transient Network Metal Coordination Hydrogels. Macromolecules.

[17] Wang C., Flynn N.T., Langer R. (2010). Controlled Structure and Properties of Thermoresponsive Nanoparticle-Hydrogel Composites. Adv. Mater..

[18] Khatyr A., Ziessel R. (2002). Stepwise Construction of Pyrene Bridged Polytopic Ligands Carrying Acetylenic Tethers. Tetrahedron Lett..

[19] Stadler A.M., Puntoriero F., Campagna S., Kyritsakas N., Welter L., Lehn J.M. (2005). Synthesis, Structural Features, Absorption Spectra, Redox Behaviour and Luminescence Properties of Ruthenium(Ii) Rack-Type Dinuclear Complexes with Ditopic, Hydrazone-Based Ligands. Chemistry.

[20] Liu P., Shi G.H., Chen X.G. (2020). Terpyridine-Containing π-Conjugated Polymers for Light-Emitting and Photovoltaic Materials. Front. Chem..

[21] Andreas W., Andreas W., Florian S., Ulrich S.S. (2011). Advances in The Field of π-conjugated 2,2′:6′,2″-Terpyridines. Chem. Soc. Rev..

[22] Yang H., Ghiassinejad S., Ruymbeke E.V., Fustin C.A. (2020). Tunable Interpenetrating Polymer Network Hydrogels Based on Dynamic Covalent Bonds and Metal–Ligand Bonds. Macromolecules.

[23] Yu X., Wang Z., Li Y., Geng L., Ren J., Feng G. (2017). Fluorescent and Electrochemical Supramolecular Coordination Polymer Hydrogels Formed from Ion-Tuned Self-Assembly of Small Bis-Terpyridine Monomer. Inorg. Chem..

[24] Xu Y., Jerca F.A., Jerca V.V., Richard H. (2020). Self-Healing and Moldable Poly (2-isopropenyl-2-oxazoline) Supramolecular Hydrogels Based on a Transient Metal Coordination Network. Macromolecules.

[25] Bekiari V., Lianos P. (2006). Photophysical Behavior of Terpyridine-Lanthanide Ion Complexes Incorporated in A Poly (N,N-Dimethylacrylamide) Hydrogel. Langmuir.

[26] Filho C.M.C., Bueno P.V.A., Matsushita A.F.Y., Vilsinski B.H., Valente A.J.M. (2020). Uncommon Sorption Mechanism of Aromatic Compounds onto Poly (Vinyl Alcohol)/Chitosan/Maleic Anhydride-β-Cyclodextrin Hydrogels. Polymers.

[27] Hennig T.L.R., Kascholke C., Hoetzel R., Hacker M.C. (2013). Reactive and Atimuli-Responsive Maleic Anhydride Containing Macromers–Multi-Functional Cross-Linkers and Building Blocks for Hydrogel Fabrication. React. Funct. Polym..

[28] Jakub H., Eva C.K., Miroslava D.S., Radka H., Jakub S., Martin H., Jiri M., Jiri H., Petr L., Roman S. (2019). Hydrogel Tissue Expanders for Stomatology. Part II. Poly (styrene-maleic anhydride) Hydrogels. Polymers.

[29] Schmidt U., Zschoche S., Werner C. (2010). Modification of Poly (Octadecene-alt-Maleic Anhydride) Films by Reaction with Functional Amines. J. Appl. Polym. Sci..

[30] Uppu D.S.S.M., Akkapeddi P., Manjunath G.B., Yarlagadda V., Hoque J., Haldar J. (2013). Polymers with Tunable Side-Chain Amphiphilicity as Non-Hemolytic Antibacterial Agents. Chem. Commun..

[31] Koning C., Ikker A., Borggreve R., Leemans L., Miller M. (1993). Reactive Blending of Poly (Styrene-co-Maleic Anhydride) with Poly (Phenylene Oxide) by Addition of (Alpha)-Amino-Polystyrene. Polymer.

[32] Hanabusa K., Nakamura A., Koyama T., Shirai H. (1992). Synthesis, Polymerization, Copolymerization, and Transition-Metal Coordination of 4-(2,2′:6′,2″-terpyridin-4′-yl) styrene and its Polymers and Copolymers. Macromol. Chem. Phys..

[33] Chen X.G., Zhou Q.G., Cheng Y.X., Geng Y.H., Ma D.G. (2007). Synthesis, Structure and Luminescence Properties of Zinc (II) Complexes with Terpyridine Derivatives as Ligand. J. Lumin..

[34] Li B., Berliner M., Buzon R., Chiu C.K.F., Colgan S.T., Kaneko T., Keene N., Kissel W., Le T., Leeman K.R. (2006). Aqueous Phosphoric Acid as a Mild Reagent for Deprotection of Tert-Butyl Carbamates, Esters, and Ethers. J. Org. Chem..

[35] Yang M., Liu B.T., Gao G., Liu X.L., Liu F.Q. (2010). Poly (Maleic Anhydride-co-Acrylic acid)/Poly (Ethylene Glycol) Hydrogels with pH- and Ionic-Strength-Responses. Chin. J. Polym. Sci..

[36] Barman S., Konai M.M., Samaddar S., Haldar J. (2019). Amino-Acid Conjugated Polymers: Antibacterial Agents Effective against Drug-resistant A. baumannii with no Detectable Resistance. ACS Appl. Mater. Interfaces.

[37] Zhou G.C., Harruna I.I. (2005). Synthesis and Characterization of Bis (2,2′:6′,2′′-Terpyridine) Ruthenium(II)-Connected Diblock Polymers via RAFT Polymerization. Macromolecules.

[38] Holyer R.H., Hubbard C.D., Kettle S.F.A., Wilkins R.G. (1965). The Kinetics of Replacement Reactions of Complexes of the Transition Metals with 1,10-Phenanthroline and 2,2′-Bipyridine. Inorg. Chem..

[39] Carlos G.S., Lohmeijer B.G.G., Meier M.A.R., Schubert U.S. (2005). Synthesis of Terpyridine-Terminated Polymers by Anionic Polymerization. Macromolecules.

[40] Zhang A.J., Chen C.J., Lou D.Y., Miao Y.H., Qiao C.X., Hu J.B. (2016). Nickel (II), Cadmium (II), and Copper (II) Complexes Based on Ditopic Terpyridine Derivative Ligand: Syntheses, Crystal Structures, and Luminescent Properties: Nickel (II), Cadmium (II), and Copper (II). Z Anorg. Allg. Chem..

[41] Naik R.M., Rai J., Srivastava A., Ratan S., Singh I. (2015). Kinetic and Mechanistic Studies of Uncatalyzed Substitution of Coordinated Cyanide in Hexacyanoferrate (II) by 2,2′-Bipyridine. Int. J. Chem. Phys. Sci..

[42] Holyer R.H., Hubbard C.D., Kettle S.F.A., Wilkins R.G. (1966). The Kinetics of Replacement Reactions of Complexes of the Transition Metals with 2,2′,2″-Terpyridine. Inorg. Chem..

[43] Zhang Y., Gu H., Yang Z., Xu B. (2003). Supramolecular Hydrogels Respond to Ligand-Receptor Interaction. J. Am. Chem. Soc..

[44] Reddy S.M.M., Augustine G., Ayyadurai N., Shanmugam G. (2018). Biocytin-Based pH-Stimuli Responsive Supramolecular Multivariant Hydrogelator for Potential Applications. ACS Appl. Bio. Mater..

[45] Li N., Liu C.J., Chen W. (2019). Facile Access to Guar Gum Based Supramolecular Hydrogels with Rapid Self-Healing Ability and Multi-Stimuli Responsive Gel-Sol Transitions. J. Agric. Food Chem..

[46] Appel E.A., Loh X.J., Jones S.T., Biedermann F., Dreiss C.A., Scherman O.A. (2012). Ultrahigh-Water-Content Supramolecular Hydrogels Exhibiting Multistimuli Responsiveness. J. Am. Chem. Soc..

[47] Song G.S., Zhao Z.Y., Peng X., He C.C., Weiss R.A., Wang H.L. (2016). Rheological Behavior of Tough PVP-In Situ-PAAm Hydrogels Physically Cross-Linked by Cooperative Hydrogen Bonding. Macromolecules.

[48] Kitazawa Y., Iwata K., Imaizumi S., Ahn H., Sung Y.K., Ueno K., Moon J.P., Watanabe M. (2014). Gelation of Solvate Ionic Liquid by Self-Assembly of Block Copolymer and Characterization as Polymer Electrolyte. Macromolecules.

[49] Miao T.X., Fenn S.L., Charron P.N., Floreani R.A. (2015). Self-Healing and Thermoresponsive Dual-Cross-Linked Alginate Hydrogels Based on Supramolecular Inclusion Complexes. Biomacromolecules.

[50] Liu J., Chen G., Guo M., Jiang M. (2010). Dual Stimuli-Responsive Supramolecular Hydrogel Based on Hybrid Inclusion Complex (HIC). Macromolecules.

[51] Sarkar K., Dastidar P. (2018). Supramolecular Hydrogel Derived from a C3-Symmetric Boronic Acid Derivative for Stimuli Responsive Release of Insulin and Doxorubicin. Langmuir.

[52] Raghavan S.R., Cipriano B.H. (2006). Gel Formation: Phase Diagrams Using Tabletop Rheology and Calorimetry. Molecular Gels.

